# Retinal Image Denoising via Bilateral Filter with a Spatial Kernel of Optimally Oriented Line Spread Function

**DOI:** 10.1155/2017/1769834

**Published:** 2017-02-05

**Authors:** Yunlong He, Yuanjie Zheng, Yanna Zhao, Yanju Ren, Jian Lian, James Gee

**Affiliations:** ^1^School of Information Science and Engineering, Shandong Normal University, Jinan 250014, China; ^2^Perelman School of Medicine, University of Pennsylvania, Philadelphia, PA 19104, USA; ^3^Institute of Life Sciences, Shandong Normal University, Jinan 250014, China; ^4^Key Laboratory of Intelligent Information Processing, Shandong Normal University, Jinan 250014, China; ^5^School of Psychology, Shandong Normal University, Jinan 250014, China

## Abstract

Filtering belongs to the most fundamental operations of retinal image processing and for which the value of the filtered image at a given location is a function of the values in a local window centered at this location. However, preserving thin retinal vessels during the filtering process is challenging due to vessels' small area and weak contrast compared to background, caused by the limited resolution of imaging and less blood flow in the vessel. In this paper, we present a novel retinal image denoising approach which is able to preserve the details of retinal vessels while effectively eliminating image noise. Specifically, our approach is carried out by determining an optimal spatial kernel for the bilateral filter, which is represented by a line spread function with an orientation and scale adjusted adaptively to the local vessel structure. Moreover, this approach can also be served as a preprocessing tool for improving the accuracy of the vessel detection technique. Experimental results show the superiority of our approach over state-of-the-art image denoising techniques such as the bilateral filter.

## 1. Introduction

Ocular diseases may evoke changes of retinal vascular structures. To diagnose them, analyzing and interpreting the characteristics of blood vessels in retinal images play an important role [1, 2]. To obtain an automatic system for assessing retinal vessels, automated vessel detection from retinal images is a fundamental step and has long been an important research topic in fields of computer vision and medical image analysis.

Given a retinal image, the performance of a vessel detection technique depends on how it deals with two fundamental challenges: image denoising where the underlying goal is to suppress noise in image and vascular detection which aims to differentiate vessels from other components of the retina. Regarding image denoising, a great variety of techniques have been proposed, including the classical Gaussian-filtering based approaches [3–6] and the edge-preserving methods [7–10]. The latter are more widely used in denoising retinal images due to their potentials in preserving the vascular structure of the retinal vessels. As a representative edge-preserving technique, bilateral filter (BLF) [7] belongs to the most popularly used techniques due to its simplicity and effectiveness. It is accomplished by combining a spatial kernel and a range kernel which measure the spatial distance and intensity difference between pixels, respectively. Regarding vascular structure detection, various techniques have been investigated, including matched filter (MF) which employs the Gaussian-shape of the cross-section of the vessel [11–13], ridge detection which relies on the ridge shape of the vessel centerlines [1, 14, 15], classification which is based on a variety of image features and machine learning algorithms [16, 17], and vesselness measures that recognize tubular vascular structures using the eigenvalues of the Hessian matrix [18–20]. Among these techniques, MF [11] and Frangi's filter (FR) [19] are two of the most widely used vessel detection methods. MF is a simple yet effective method to detect the retinal vessels by using a Gaussian-like kernel. In essence, the intensity distribution of this kernel can be mathematically expressed as an oriented line spread function (LSF) that spreads as a Gaussian function in the theory of imaging systems [21–23]. FR performs well in detecting the tube-like structure from other components by measuring the eigenvalues of Hessian matrix computed at each pixel of a retinal image.

Disregarding the good results reported in the related works, accurate detection of retinal vessels remains an open challenge. The main reason comes from the fact that retinal vessels can be ruined during image denoising process due to the introduced negative effects of blur or vessel-structure corruption. As widely known, blur occurs when classical Gaussian based filtering is performed on the image. The edge-preserving image denoising methods (e.g., the BLF) are capable of keeping crisp edges. However, most of them may fail in maintaining retinal vessels because they ignore the special tube-like structure of the retinal vessels. This can happen especially for thin vessels due to their weak contrast and small area relative to the background and the difference in their spatial distribution compared with a common crisp edge.

In this paper, we propose a novel image filtering approach specialized for denoising retinal images while preserving the vascular structures. Our approach is realized by determining a set of spatial kernels for the BLF algorithm, which are represented by an LSF with an orientation and scale adjusted adaptively to the local vessel structures. Our LSF function is similar to the functions used in MF. However, we incorporate it in BLF for image denoising, unlike the vessel detection purpose in MF. By combining the benefits of BLF and MF, our approach is not only noniterative, simple, and easy to implement, but also able to outperform the state-of-the-art image filtering methods in maintaining details of retinal vessels and removing image noise. The advantages of our approach are validated by the improved vessel detection accuracies as shown in our experimental results.

## 2. Related Work

This section provides an overview of the current state-of-the-art techniques for both image denoising and vessel detection.

### 2.1. Image Denoising

In recent years, a large variety of approaches have been presented for image denoising. These approaches can be basically classified into two categories: classical Gaussian-filtering based techniques which may lose the edge information recognized as the primary feature of an image [3–6] and edge-preserving based methods which attempt to preserve prominent edges while denoising [7–10]. Gaussian-filtering based techniques are commonly used in medical image analysis. However, as the weights of a Gaussian filter purity depend on the spatial distance, these approaches may lose prominent image edges and introduce blurring effect, which can cause troubles to vessel detection in retinal images. Edge-preserving methods were proposed to overcome the loss of prominent edges. For example, the anisotropic diffusion filter [8] and the weighted least squares filter [9] attempt to smooth images while preserving edges based on measuring the image gradient. The nonlocal means filter [10] computes filtered result relying on the similarity of intensity and the order of pixel in their neighborhoods. BLF is distinguished for its edge-preserving ability, for which a spatial kernel and a range kernel are combined and the output at each pixel relies both on the spatial distance and intensity differences [7].

Among those edge-preserving image denoising techniques, BLF is perhaps the most widely used one due to several of its strengths. First, BLF employs a computation of weighted average, which is easy to implement. Second, BLF is a noniterative and local approach, requiring less computational cost in contrast to other iterative [8, 9] and global [10] edge-preserving methods. Third, it has been validated that BLF can keep the crisp edges while removing noise in the image. BLF has been applied to a large variety of tasks including image enhancement [24], artistic rendering [25], image editing [26], optical flow estimation [27, 28], feature recognition [29], medical image denoising [30, 31], and 3D Optical Coherence Tomography retinal layer segmentation [32].

Despite the strengths of BLF, when the task refers to retinal image denoising, BLF might be degraded because it does not take the special tube-like structure of the retinal vessels into consideration. In most image denoising scenarios, BLF tends to preserve crisp edges when two special characteristics exist: prominent contrast observed at the vicinity of edge and larger area occupied by the edge structure compared with isolated noise. In contrast, thin vessels in a retinal image are different from a common crisp edge, due to their weak contrast compared to background and tiny area in the image. BLF cannot handle these special properties of vessels and therefore details of vessels would probably be missed in the filtered image. In this paper, we present a novel BLF algorithm specialized for retinal image denoising based on the properties of the vascular structures. It outperforms the original BLF method in preserving thin vessels.

### 2.2. Vessel Detection

Recent years have witnessed several classes of approaches for retinal vessel detection and most of them depend on the special characteristics of the vascular structures. Among these approaches, matched filter approaches employ an observation that the cross-section of a retinal vessel can be approximated by a Gaussian function [11–13], ridge detection methods rely on an extraction of image ridges which can be treated as vessel centerlines [1, 14, 15], classification methods utilize a variety of image features to classify each pixel by using machine learning algorithms (e.g., a Bayesian classifier) [16, 17], and vesselness measures analyze the eigenvalues of Hessian matrix at each pixel in order to recognize tubular vascular structures [18–20].

Compared with other vessel detection methods, MF [11] is simple yet effective. Its advantages originate from the Gaussian-like kernels used in its detection process. Blood vessels are characterized by having a tubular structure with a small curvature and being darker/brighter compared with the background. These special properties can be well captured by MF, leading to a good performance in detecting thin vessels within a low-contrast region [33]. As a representative method for measuring vesselness, FR [19] is a practical and state-of-the-art technique. It exploits the eigendecomposition of the Hessian matrix to discriminate the tubular structure from other components.

Different from MF and FR which aim to detect blood vessels from retinal images, our proposed method is designed to denoise retinal images while preserving the vascular structure. It also can be applied to improve the accuracy of the vessel detection techniques.

## 3. Bilateral Filter and Matched Filter

In this section, we introduce the basic concepts of bilateral filter (BLF) [7] and matched filter (MF) [11] for convenience of describing our method in Section 4.

### 3.1. Bilateral Filter

As a nonlinear, edge-preserving image filtering method, BLF treats the intensity value at each pixel as a weighted average of its nearby pixels' intensity values [7]. BLF is capable of fixing the problem of Gaussian blur in traditional Gaussian-convolution based image filtering methods as it combines two components: Euclidean distance and radiometric difference expressed by the following equations:

(1) Dp=kp−1∑q∈RPWsdpqWrfpqIq,

(2) kp=∑q∈RPWsdpqWrfpq,

where *D*(*p*) and *I*(*q*) denote the image intensities of pixel *p* in the output image and pixel *q* in the input image, respectively. *R*_*p*_ represents a set of pixels neighboring to pixel *p*. *W*_*s*_ and *W*_*r*_ are the spatial kernel and range kernel, for which the weights are computed from the Euclidean distance *d*_*pq*_ and the photometric difference *f*_*pq*_ between pixels *p* and *q*, respectively. The latter is usually measured by image features such as intensity or texture [34, 35]. *k*_*p*_^−1^ is a normalization term computed by (2). In (1), *W*_*s*_ and *W*_*r*_ both take a value inverse to the corresponding input and are expressed typically as a Gaussian function. As an example, *W*_*s*_ is calculated by

(3) $$\begin{array}{c} W_{s}\left(\;d_{pq}\;\right)=\exp\left(\;-\frac{d_{pq}^{2}}{2\sigma_{s}^{2}}\;\right). \end{array}$$

In (3), *σ*_*s*_ is a scale parameter determining the weight distribution pattern of the kernel. A large *σ*_*s*_ means that the range Gaussian widens and flattens.

BLF outperforms many other image filtering algorithms due to its ability to achieve good filtering behavior while preserving crisp edges [7]. It is obtained by combining the spatial kernel and the range kernel in (1). In smooth regions, BLF performs as a Gaussian low-pass filter by averaging away the small, weakly correlated differences between pixel values caused by noise, thanks to the spatial kernel. For a sharp boundary formed by a dark region and a bright one, BLF replaces the dark pixels by an average of the dark pixels in its vicinity while ignoring bright pixels and* vice versa*, thanks to the range kernel.

However, it is not trivial for BLF to distinguish between thin vessels and noise when applying it to retinal images. This is caused by several characteristics of the specific structure of thin vessel compared with a common image edge which is formed by dark and bright regions. First, the pixels of the thin vessel occupy a smaller portion of the pixels in its local window, causing the vessel pixels to be averaged away by the spatial kernel. Second, image intensities of thin vessels are likely to be close to the background due to the limited resolution of retinal imaging and less blood flow, causing vessel pixels to be averaged away by the range kernel. Third, the spatial distribution of vessel pixels is significantly different from independent, isolated image noise, but BLF lacks functions to fully capture the related specific properties.

### 3.2. Matched Filter

The matched filter (MF) [11] is a vessel detection method based on the observation that the cross-section of vessels can be modeled as a Gaussian function. In this method, a given retinal image is convolved with a set of Gaussian-like kernels built in different orientations, then taking the maximum response over all orientations as the result at each pixel. Since blood vessels have lower reflectance compared with other retinal tissues [36, 37], the vessels appear darker than the background in the retinal image. Yet many techniques also work on the inverted retinal images [17, 38], in which the vessels may appear brighter than the background. MF is valid for both the darker and brighter cases.

Assume the blood vessels appear darker than the background. Let *n* denote the number of orientations. The *i*th oriented kernel *K*_*i*_ matched to a vessel at an angle *θ*_*i*_ can be mathematically defined as

(4) Kix,y=−exp⁡−u22σ2for  v≤L2, i∈1,2,3⋯n,

(5) uv=xycos⁡θisin⁡θi−sin⁡θicos⁡θi,

where (*x*, *y*) are the coordinates with the origin being at the center of the kernel window and (*u*, *v*) are the new coordinates computed by (5) at an angle *θ*_*i*_. *σ* is the variance used to control the scale of this Gaussian function, which can be set to different values to detect both thin and thick vessels in its extended methods [13]. *L* denotes the length of the kernel window. In essence, each value in this kernel depends on the perpendicular distance between the point (*x*, *y*) and an oriented straight line passing through the center of the vessel. In particular, when *θ*_*i*_ = 0°, the coordinate *u* = *x* and the direction of the vessel is aligned with the *y*-axis and can be expressed as

(6) $$\begin{array}{c} K\left(\;x,y\;\right)=-exp\left(\;-\frac{x^{2}}{2\sigma^{2}}\;\right)\;for\;\;\left|y\right|\leq \frac{L}{2}. \end{array}$$

This equation demonstrates the means to detect the vessels distributed in the vertical direction. To deal with vessels appearing brighter than the background, the value of the above kernels should be inverted accordingly (e.g., take the absolute value of (4)).

Unlike the spatial kernel computed by a common Gaussian function in (3), the MF kernel uses a particular model. Indeed, in the area of imaging systems, this model can be mathematically expressed as a particular LSF [21–23] that spreads as a Gaussian function. The intensities of this particular LSF are distributed equally on one orientation with a Gaussian profile. It can model the orientation and the cross-section of the vascular structure very well. Moreover, the scale parameter *σ* can also be adjusted for different vascular widths. Therefore, even for thin vessels with low contrast, MF provides the best overall response compared with most techniques [33].

## 4. Our Method

The common spatial kernel (as expressed in (3)) of BLF is isotropic across the whole image and we find that adjusting this kernel according to the local vessel structure can significantly improve the performance of image denoising. A blood vessel appears locally in the retinal image as a dark/bright tube with a small curvature, and the shape of its cross-section is similar to a Gaussian function. These properties can be represented by an LSF that spreads as a Gaussian function. Therefore, we propose to determine the spatial kernel of BLF by a particular LSF, which can be obtained by using the Gaussian-like kernel of MF. The proposed new BLF performs significantly better in preserving thin blood vessels in retinal images while denoising image. Moreover, with some simple experiments, we find it outperforms the original BLF in some other aspects that benefit vascular structure preservation.

### 4.1. Bilateral Filtering with a Spatial Kernel of Optimally Oriented LSF

Given a retinal image, our filter also computes a weighted average of the local pixels based on (1). The difference lies in the definition of the spatial kernel *W*_*s*_. Mathematically, the method can be expressed as

(7) Dp=kp−1∑q∈RPWs′dvqWrfpqIq,kp=∑q∈RPWs′dvqWrfpq,

where *W*_*s*_′ is the proposed spatial kernel which is adaptive to different local orientations and scales of the blood vessels. The value of *W*_*s*_′ is determined by *d*_*vq*_. We can obtain *d*_*vq*_ by computing the perpendicular distance between pixel *p* and the straight line passing through the center of blood vessel *v*, rather than measuring the Euclidean distance between different pixels by the spatial kernel of BLF. *k*_*p*_ is also a normalization term. The core idea of our method is to design an optimal spatial kernel *W*_*s*_′ and combine it with the range kernel of BLF.

In order to obtain *W*_*s*_′, we start with constructing several multiscale oriented Gaussian-like kernels based on MF. For each pixel at its local window in a retinal image, we use these Gaussian-like kernels to detect the vessel's orientation and scale. The best matching one is selected as the detection result. *W*_*s*_′ at each pixel can be simply computed by using the detection result. Finally, *W*_*s*_′ combined with the range kernel results in our filter.

#### 4.1.1. Multiscale Oriented Gaussian-Like Kernels

To construct the multiscale oriented Gaussian-like kernels, the equations of MF are used. The process is similar to that in MF: compute one case aligned with the *y*-axis using (6); then rotate it by (5) to generate *n* oriented Gaussian-like kernels. Moreover, in order to capture vessels at a variety of widths, we apply the oriented Gaussian-like kernels at different scales; that is, we further set *m* different scale parameters for each orientation. Then we can obtain a total of *m* · *n* multiscale oriented Gaussian-like kernels. Suppose that the vessels appear darker than the background in the retinal image; one of these kernels is defined as

(8) Ki,j′x,y=−exp−u22σj2for  v≤L2,  i∈1,2,3⋯n,  j∈1,2,3⋯m,

where *K*_*i*,*j*_′ denotes the *i*th oriented Gaussian-like kernel in the *j*th scale with the parameters *θ*_*i*_ and *σ*_*j*_. (*u*, *v*) can be computed using (5) at an angle *θ*_*i*_. When the vessel is brighter than the background in retinal image, the value of *K*_*i*,*j*_′ needs to be inverted. The calculating process is not computationally expensive since these kernels can be calculated independently of the retinal image without relying on the image intensity.

#### 4.1.2. Local Vessel Detection

**Figure 1:**
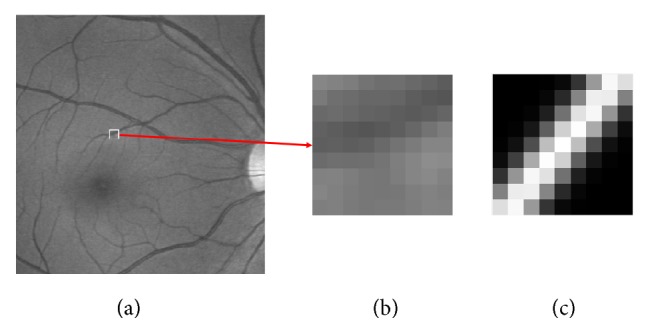
The spatial kernel (a) determined by our algorithm for the center pixel of a local window (b) chosen from a retinal image (c). Obviously, our kernel has an orientation and scale consistent with the corresponding retinal vessel.

Given a retinal image, the aim of local vessel detection is to detect the vessel's orientation and the scale of each pixel at its local window. In this process, the retinal image is convolved with the *m* · *n* multiscale oriented Gaussian-like kernels constructed in advance and then each point has *m* · *n* responses. Then we select the one with the maximum response as the detecting result at each point. The detecting process can be expressed by the following:

(9) Kt,s′=arg⁡maxKi,j′Ki,j′·Fpi,t∈1,2,3⋯n,  j,s∈1,2,3⋯m,

where *K*_*t*,*s*_′ is the kernel selected from *m* · *n* different multiscale oriented Gaussian-like kernels which has the maximum response. *F*(*p*) is the intensity matrix of local window centered at pixel *p*. *t* and *s* are the index numbers of *n* orientations and *m* scales. Symbol · in (9) represents the dot product of two matrices. All of the kernels and intensity matrices need to be normalized before computing. An exampling spatial kernel specified by our approach is shown in Figure 1.

#### 4.1.3. Computation of Spatial Kernel

Then we use the detection result to get *W*_*s*_′ at each pixel. When the retinal vessels are darker than the background, the value of the normalized detection result *K*_*t*,s_′ is negative and varies from −1 to 0. When the retinal vessels are brighter than the background, the value of *K*_*t*,*s*_′ varies from 0 to 1. In order to obtain the weights *W*_*s*_′ from the value of *K*_*t*,*s*_′ in both of the two cases, we set (*x*, *y*) as the coordinate of pixel *q* in the retinal image, and then the weight of each pixel *q* can be obtained using the following:

(10) $$\begin{array}{c} W_{s}^{\prime }\left(\;d_{vq}\;\right)=\left|\;K_{t,s}^{\prime }\left(\;x,y\;\right)\;\right|. \end{array}$$

Expand the coordinates term *u* and *K*_*t*,*s*_′(*x*, *y*) using (5) and (8), and the weight of each pixel can be computed by

**Figure 2:**
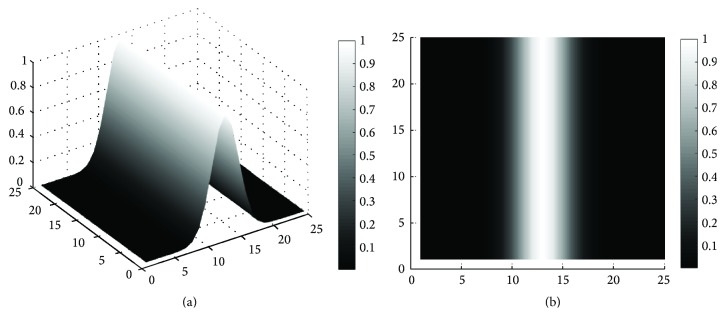
Weight distribution of the proposed spatial kernel, as demonstrated by a 3D shaded surface plot (a) and 2D gray image display (b).

(11) Ws′dvq=exp⁡−xcos⁡θt−ysin⁡θt22σs2t∈1,2,3⋯n,  s∈1,2,3⋯m,

where *θ*_*t*_ and *σ*_*s*_ represent the orientation and scale of the proposed kernel which has a maximum response. The weight distribution of this spatial kernel is adaptive to the vessel's orientation and scale, which is different from the common spatial kernel used in BLF.  Figure 2 shows the proposed spatial kernel aligned with the *y*-axis used at one scale.

#### 4.1.4. Optimally Oriented LSF for BLF

**Figure 3:**
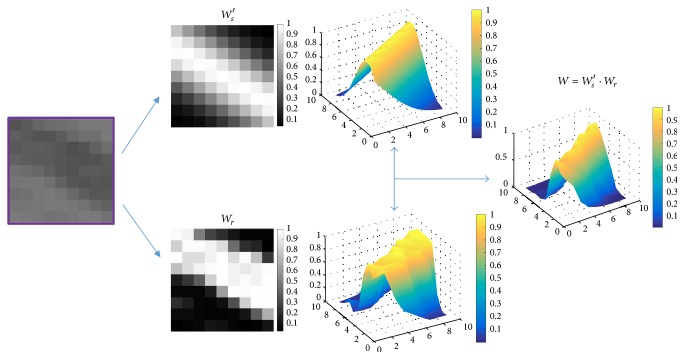
Our image filter combines a proposed spatial kernel *W*_*s*_′ and a range kernel *W*_*r*_. From left to right: an image patch bearing vessel, gray image display of the kernels, 3D shaded surface plot of the kernels and 3D shaded surface plot of the final kernel, respectively.

Finally, we combine *W*_*s*_′ with the range kernel using (7) to get the new filter.  Figure 3 illustrates the incorporating process of the proposed spatial kernel *W*_*s*_′ and the range kernel *W*_*r*_.

It is worth noting that the proposed special kernel *W*_*s*_′ provides an optimal scheme for retinal image denoising. The intensity distribution of the proposed spatial kernel can be mathematically defined as an LSF that spreads as a Gaussian function. The weight distribution of the proposed special kernel with this LSF model provides more advantages for image filtering. First, weights in this kernel are distributed equally on a oriented line and reach their maximum value on the center line. This property corresponds with the vessel's tube-like structure and is able to give large weights to the vessel pixels. Second, a Gaussian profile retains the property of Euclidean distance which attempts to protect the local structures from being corrupted by the nonvessel pixels from the vertical direction. Third, it combines the advantages of both BLF and MF, including being noniterative, local, easy to implement, and effective in capturing thin vessels. Moreover, this novel BLF can also be adjusted by two parameters; hence it is a spatial kernel of an optimally oriented LSF.

### 4.2. Discussions of Parameters

There are five adjustable parameters in our method: *k* (kernel size), *σ*_*s*_ (variance of spatial kernel), *σ*_*r*_ (variance of range kernel), *m* (number of vessels' scales), and *n* (number of orientations). *k* is the kernel size used in both vessel detection and image filtering, which should be set according to the vessel thickness in retinal images. *σ*_*s*_ and *σ*_*r*_ are the scale parameters of our spatial kernel and the range kernel, which are used to adjust the degree of blurring based on novel spatial distribution and intensity similarity. A larger *σ*_*s*_ or *σ*_*r*_ can remove noise more effectively, at the cost of losing more thin vessels. *n* is the number of spatial kernels, which depends on the practical experimental data. All these parameters should be set according to specific task. In our experiments, we empirically set *k* = 9, *σ*_*s*_ = 0.4,0.8,1.2,1.6, *σ*_*r*_ = 0.04, *m* = 4, and *n* = 12.

### 4.3. Implementation Details

The proposed algorithm is implemented in Matlab and executed on a 3.30 GHz Intel® Core™ i5-4590 processor with 8 GB RAM. The structure of the program can be divided into two parts: kernel determination and image filtering. For each pixel *p* in retinal image, kernel determination aims to specify an optimal kernel with the best matching orientation and scale. For image filtering, each pixel in the filtered image is computed by using (7). A pseudocode example of our algorithm is illustrated in Algorithm 1.

## 5. Evaluation Metrics

In order to quantitatively measure the performance of the proposed method, the denoised image is converted into a binary image and then compared with the corresponding ground truth. The ground truth is generated by manually marking each pixel as either vessel or background. Five commonly used quantitative evaluation metrics [2, 36, 39] are employed, including sensitivity (Se), specificity (Sp), accuracy (Acc), the area under a receiver operating characteristic curve (AUC), and the Dice coefficient (DC). These metrics are defined as follows:

(12) Se=tptp+fn,Sp=tntn+fp,Acc=tp+fntp+fp+tn+fn,AUC=Se+Sp2,DC=2A∩BA+B,

where *tp*, *tn*, *fp*, and *fn* represent the number of correctly identified vessel pixels, correctly identified nonvessel pixels, incorrectly identified vessel pixels, and incorrectly identified background pixels, respectively. Se and Sp are used to measure the ratio of correctly identified vessel and nonvessel pixels, and Acc indicates the ratio of the total correctly identified pixels [36]. As the vessel pixels of a retinal image are often much fewer than the background pixels, the value of Acc is mainly determined by the background. In this case, another performance metric AUC suggested by [39] should be a good choice to obtain a balance between vessel and background. In addition, DC is also a widely used metric. It is computed as the overlapping area between the ground truth *A* and the resulting binary image *B* [2].

## 6. Experimental Results

In this section, we show the results of our experiments conducted to evaluate our method both qualitatively and quantitatively. We compared our method with BLF as BLF is a representative image filtering technique. Our comparisons are based on our synthetic vessel images and the public database of Digital Retinal Images for Vessel Extraction (DRIVE) [1].

### 6.1. Synthetic Vessel Images

To validate the proposed image filter, we created 8 synthetic images in which the vessels are manually drawn with the “Paint” tool in the Microsoft Windows system. These synthetic vessels are characterized by straight lines with different thicknesses and bifurcated vessel structures (as shown in Figure 5). In these images, vessels and background pixels were assigned an intensity value of 180 and 125, respectively. Image noises of Gaussian and salt-and-pepper were added to these images.

### 6.2. DRIVE Database

The DRIVE database (http://www.isi.uu.nl/Research/Databases/DRIVE/) contains a total of 40 RGB color retinal images. These retinal images were captured by a Canon CR5 nonmydriatic 3-CCD camera (Canon, Tokyo, Japan) with a 45-degree field of view (FOV). The size of each image is 768 × 584 pixels. For all these images, manual marks of the vessels are provided and were treated as the ground truth for vessel detection in our experiments. Our experiments were conducted on the green channel of the original RGB retinal images because it preserves the largest vessel-background contrast, as described in [36, 40, 41].

### 6.3. Qualitative Evaluation

#### 6.3.1. Synthetic Vessel Images

We compared our method with BLF by observing their results generated from the synthetic vessel images and found that our method outperforms BLF in the following four aspects.

**Figure 4:**
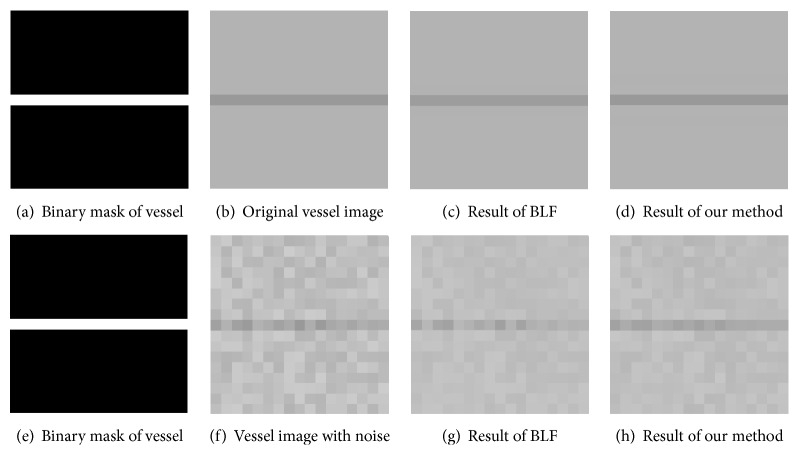
Our approach can suppress image noise while keeping vessels, as shown by a comparison of the denoising results on an artificial vessel image with BLF.

*Preserving Thin Vessels.* One of the key requirements of an ideal retinal image filtering method lies in the preservation of the thin vessels during the denoising process. As shown by the results in Figure 4, our method is able to better preserve thin vessels while achieving an effective image denoising than the standard BLF algorithm. This is attributed to the capability of our spatial kernel to emphasize the vessel pixels more when computing the weighted average as explained above.

**Figure 5:**
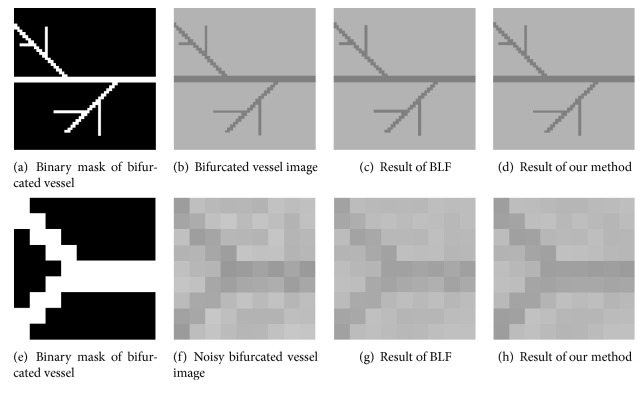
Our approach can preserve the structure of bifurcated vascular when denoising the image, as shown by the comparison of denoising results with BLF on image without noise (a–d) and with noise (e–h).

*Maintaining Bifurcated Vascular Structure.* As shown in Figure 5, the bifurcated vascular structure is maintained better by our method than the BLF algorithm during the image denoising process. This results from the fact that our method combines an adaptive spatial kernel with a range kernel, which introduces a better robustness while keeping the advantage of the original BLF algorithm.

**Figure 6:**
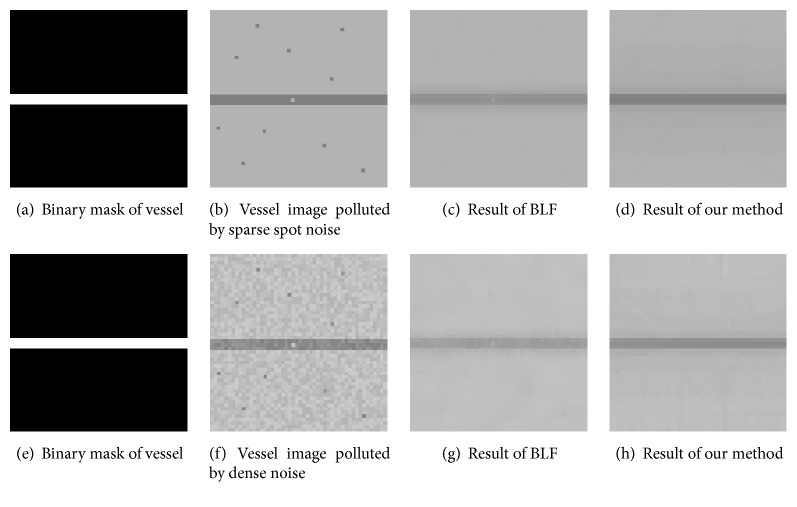
Our approach can preserve vessel when denoising image whether the image bears sparse spot noise or dense noise.

*Denoising.* Image denoising is helpful for enhancing the accuracy of vessel detection. As shown by the results in Figure 6 from the synthetic vessel images contaminated by Gaussian noise plus salt-and-pepper noise, our method is able to eliminate noise on the vessel while keeping the vascular structure. In contrast to the BLF algorithm, it benefits from the proposed spatial kernel. 

**Figure 7:**
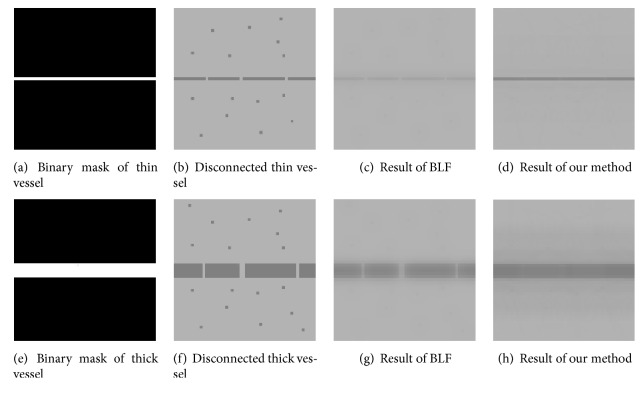
Our approach performs much better than BLF in connecting vessels separated by noise and keeping thin vessels during the image denoising process.

*Connecting Broken Vessels.* Our method is also able to connect broken vessels, which can be seen from the filtering results in Figure 7 obtained from images of synthetically disconnected vessels.

#### 6.3.2. Real Retinal Images

**Figure 8:**
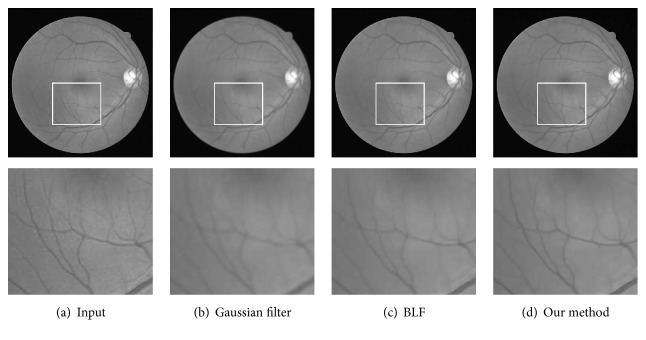
Comparison of results in retinal image denoising. Top: the original image and the denoised ones with different methods. Bottom: enlarged view of an area enclosed by a local window.

Another qualitative evaluation is based on the observation that a good retinal image filtering method should not blur/ruin the vascular structures while eliminating noise. In Figure 8, we show the green channel of a retinal image randomly chosen from the DRIVE database and the results obtained using three image denoising algorithms including Gaussian filter, BLF, and our method. From the results, we can see that the classical Gaussian filter causes blur at the vicinity of the vascular structure. By contrast, BLF performs better in capturing the crisp vascular structure. However, when compared with our method, thin vessels with low image contrast are corrupted in the process of image denoising of BLF. Obviously, our method generates results superior to both the Gaussian filter and BLF especially in preserving thin vessels.

### 6.4. Quantitative Evaluation

#### 6.4.1. Synthetic Vessel Images

In order to further confirm the merits of our method, we carried out quantitative comparisons by using the synthetic vessel images. We ran the BLF algorithm and our method on these images and computed the values of Se, Sp, Acc, AUC, and DC by comparing the algorithms' results with the ground truth. As shown in Table 1, our method behaves better than BLF in preserving thin vessels even if noise exists in the image.

The experimental results obtained from images in Figure 5 are presented in Table 2. From Figure 5 we can observe that our method can preserve the complicated bifurcate vessel structures well, whether the images are contaminated by noise or not. In contrast, the results of BLF is degraded significantly by the image noise.

The advantages of our algorithm are also reflected in its good performances in dealing with the image noise regarding vessel pixels. As shown in Table 3, our method outperforms BLF in denoising the vessel parts of the images, whether Gaussian noise or salt-and-pepper noise is added. This results from the introduced spatial kernel which can adaptively weight more the vessel pixels when computing the weighted average within a local window.

Table 4 contains performance of our method and BLF in connecting discontinuous vessels, which further confirms the merit of our method. As shown in Table 4, our method outperforms BLF on images with both disconnected thin vessels and thick vessels. It can also be observed that BLF performs worse on thin vessel images than on thick vessel images. This indicates that the performance of BLF is unstable when the scale of the vessels changes.

#### 6.4.2. Real Retinal Images

**Figure 9:**
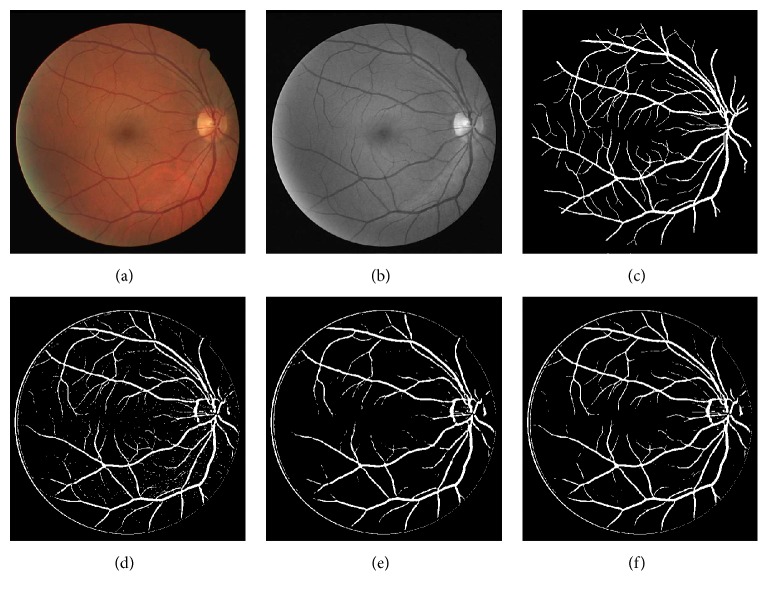
Vessel detection results with different strategies. (a) Original retinal image. (b) Green channel image of (a). (c) Ground truth vascular structures. (d) Vessel detection result of Frangi's filter. (e) Vessel detection result of BLF and Frangi's filter. (f) Vessel detection result of our method and Frangi's filter.

In the following experiments, we took advantage of the images from the DRIVE database to demonstrate that the proposed method is able to improve the accuracy of vessel detection. To carry out the experiment, the state-of-the-art vessel detection method FR [19] was adopted. We ran BLF and our method as a preprocessing step for the vessel detection process. The detection results of different strategies are given in Figure 9. From these results, we can see that FR's results still contain a few isolated points (pixels belong to background as shown in Figure 9(c)). These points are caused by the isolated noise in the original image. To tackle this problem, we use BLF to reduce the isolated noise in the original retinal image before vessel detection.  Figure 9(e) gives the result of this strategy, which shows that the noise has been reduced. However, a large amount of thin vessels also disappeared, which degrades the quality of vessel detection.  Table 5 gives a detailed quantitative analysis of Figures 9(d) and 9(e). Overall, combining BLF with FR does not improve the detection result. In contrast, when combined with our space kernel, FR achieves the best performance. The isolated points in Figure 9(f) are much fewer than the ones in Figure 9(d) and the best values of Sp, Acc, AUC, and DC were generated by our method as shown in Table 5.

## 7. Conclusions and Future Work

In this paper, we propose a novel image filtering approach specialized for detecting vessels from retinal images. Different from existing techniques, the proposed approach leverages the special characteristics of vascular structures and determines a set of BLF spatial kernels that are oriented and scaled optimally. In addition, our spatial kernels replace the traditional Euclidean distance between pixels in the original BLF algorithm with the perpendicular distance between the point and the straight line passing through the center of the blood vessel. Our image filtering technique combines the benefits of both BLF and MF, resulting in a better performance than state-of-the-art techniques in detecting and preserving the thin vascular structures as validated by our experiments.

Our future work would include an application of our method on retinal images from patients in order to validate its performance on images with vasculature variations caused by ocular diseases. Moreover, we think our approach works as well on other tabular structures (e.g., pulmonary fissure in 15 CT images). We will assess this performance in our future work.

## Figures and Tables

**Algorithm 1:**
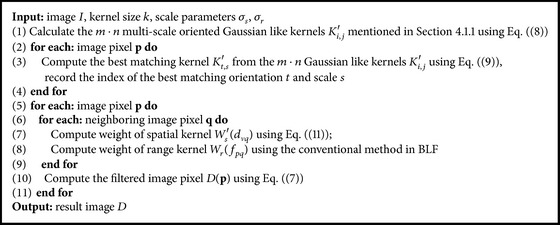
Bilateral filter with a spatial kernel of optimally oriented line spread function.

**Table 1:** Quantitative results in preserving thin vessels.

Image in Figure 4	Se	Sp	Acc	AUC	DC
(b) Thin vessel image	1	1	1	1	1
(c) Result of BLF	0.973	0.998	0.992	0.986	0.910
(d) Result of our method	0.989	0.999	0.996	0.994	0.951

(j) Noisy vessel image	0.986	0.993	0.986	0.993	0.993
(k) Result of BLF	0.961	0.993	0.962	0.977	0.980
(l) Result of our method	0.976	0.993	0.995	0.985	0.990

**Table 2:** Quantitative results in preserving bifurcated vascular structure.

Image in Figure 5	Se	Sp	Acc	AUC	DC
(b) Bifurcated vessel image	1	1	1	1	1
(c) Result of BLF	0.871	0.994	0.993	0.935	0.925
(d) Result of our method	0.933	0.998	0.951	0.966	0.966

(f) Noisy bifurcated vessel image	0.836	0.998	0.864	0.918	0.911
(g) Result of BLF	0.812	0.998	0.840	0.906	0.896
(h) Result of our method	0.908	0.998	0.955	0.953	0.948

**Table 3:** Quantitative results in retinal vessel denoising.

Image in Figure 6	Se	Sp	Acc	AUC	DC
(b)Vessel image polluted by sparse spot noise	0.998	0.937	0.996	0.968	0.997
(c) Result of BLF	0.997	0.995	0.993	0.996	0.995
(d) Result of our method	0.998	0.997	0.994	0.998	0.998

(f) Vessel image polluted by dense noise	0.980	0.927	0.978	0.954	0.988
(g) Result of BLF	0.957	0.991	0.958	0.974	0.977
(h) Result of our method	0.998	0.995	0.995	0.997	0.981

**Table 4:** Quantitative results of vessel connection.

Image in Figure 7	Se	Sp	Acc	AUC	DC
(b) Disconnected thin vessel	0.999	0.758	0.993	0.878	0.996
(c) Result of BLF	0.982	0.994	0.999	0.988	0.976
(d) Result of our method	0.993	0.997	0.992	0.995	0.981

(f) Disconnected thick vessel	0.991	0.931	0.985	0.961	0.992
(g) Result of BLF	0.992	0.978	0.993	0.985	0.976
(h) Result of our method	0.998	0.996	0.985	0.997	0.997

**Table 5:** Quantitative results of vessel detection using different strategies.

Image in Figure 9	Se	Sp	Acc	AUC	DC
(d)	0.792	0.977	0.962	0.884	0.766
(e)	0.807	0.970	0.959	0.889	0.731
(f)	0.806	0.977	0.964	0.891	0.773
